# Titanium and Zirconium Levels Are Associated with Changes in MicroRNAs Expression: Results from a Human Cross-Sectional Study on Obese Population

**DOI:** 10.1371/journal.pone.0161916

**Published:** 2016-09-09

**Authors:** Gianguido Cossellu, Valeria Motta, Laura Dioni, Laura Angelici, Luisella Vigna, Giampietro Farronato, Angela Cecilia Pesatori, Valentina Bollati

**Affiliations:** 1 Department of Biomedical, Surgical and Dental Sciences, Fondazione IRCCS Cà Granda, Ospedale Maggiore Policlinico, Università degli Studi di Milano, Via della Commenda 10, 20122, Milan, Italy; 2 EPIGET - Epidemiology, Epigenetics and Toxicology Lab, Department of Clinical Sciences and Community Health, Università degli Studi di Milano, Milan, Italy; 3 Worker’s Health Protection and Promotion Unit, Fondazione IRCCS Ca’ Granda Ospedale Maggiore Policlinico, Milan, Italy; 4 Epidemiology Unit, Fondazione IRCCS Ca’ Granda Ospedale Maggiore Policlinico, Milan, Italy; University of Massachusetts Medical School, UNITED STATES

## Abstract

**Objectives:**

In this study on 90 individuals we aimed at evaluating the microRNAs (miRNAs) expression profile associated with personal levels of Titanium (Ti) and Zirconium (Zr) traced in hair samples. Ti and Zr materials are broadly used for dental implants but the biological reactions triggered by a long term presence of these materials in the oral cavity still need to be assessed. MiRNAs are mechanisms that need to be investigated as they play a fundamental role in the control of gene expression following external stimuli and contribute to a wide range of pathophysiological processes.

**Methods:**

Using the TaqMan^®^ Low-Density Array, we assessed the expression levels of 377 human miRNAs in peripheral blood of 90 subjects. Hair samples were analyzed for Ti and Zr content using Inductively Coupled Plasma-Mass Spectrometry. We performed multivariable regression analysis to investigate the effects of Ti and Zr exposure on miRNA expression levels. We used the Ingenuity Pathway Analysis (IPA) software to explore the functional role of the investigated miRNAs and the related target genes.

**Results:**

Seven miRNAs (miR-99b, miR-142-5p, miR-152, miR-193a-5p, miR-323-3p, miR-335, miR-494) resulted specifically associated with Zr levels. The functional target analysis showed that miRNAs are involved in mechanisms such as inflammation, skeletal and connective tissue disorders.

**Conclusions:**

Our data suggest that Zr is more bioactive than Ti and show that miRNAs are relevant molecular mechanisms sensitive to Zr exposure.

## Introduction

Over the last few decades there has been an increasing interest in studying the effects of exposure to exogenous elements on several health effects such as developmental disorders, endocrine disruption, immunological syndromes, different types of cancers and even death [1]. Although human exposure to exogenous elements is often occupational, due to the high industrial use, the attention is moving towards non-occupational environments. The study of biomedical application in dentistry raised interest, as materials like Titanium (Ti) and Zirconium (Zr) are broadly used in prostheses, implants and orthodontic applications. These elements haven't any biological function and their presence in human body may reflect not only inhalation, ingestion, or skin absorption [2, 3], but also the release from the metallic implant surface of biomedical devices due to electrochemical dissolution, frictional wear, or a synergistic combination of the two.

Titanium has been the material of choice for dental implants for about 30 years and the success rates for various indications have been persistently high during the past decade. Yet the development of new materials was promoted due to some disadvantages of titanium implants, such as the unfavorable aesthetic results related to Ti shining through or the noticeable metallic portion in case of gingival recession. For these reasons new dental ceramic materials such as Zr-based ceramics have been successfully introduced and widely used in the clinic [4–6]. Zirconium oxide (ZO_2_) is a bio-inert material that exhibits high mechanical strength, excellent corrosion resistance and good biocompatibility. The characteristics of tooth-colour like, the ability to be machined and the low plaque affinity make Zr the material of choice in the esthetic of the oral cavity, especially compared with Ti devices [7, 8].

Many clinical, in-vitro and experimental animal studies, focused on the potential health effects related to Ti and Zr, mainly showed a lack of negative consequences [9–12]. However, Ti and Zr materials used in dentistry stay in close contact with the surrounding tissues for a long time. all these metallic materials used in surgery and dentistry that stay permanently in the tissues are liable, to a certain degree, to corrosion due to variations in the internal electrolyte milieu [13]. When metal particles and ions are released from the implant surface, they can migrate systemically, remain in the intercellular spaces near the site where they were released, or being up-taken by macrophages [14, 15]. It has been shown that, due to their nature, Ti and Zr can release ions that are able to cross the cell membrane triggering the production of reactive oxygen species (ROS) [16], leading to cytotoxicity, oxidative damage, and direct binding to lipids, proteins and DNA [17–19].

The mechanisms through which exogenous elements cause toxic effects seems to be also strictly related to epigenetic alterations, mitotically and meiotically heritable changes in gene expression that do not involve mutation the DNA sequence [20]. Among epigenetic changes, microRNAs (miRNAs) represent an attractive and promising mechanism to be investigated. MiRNAs are single-stranded RNAs of ~22 nt that can regulate hundreds of target genes [21]. Recent experimental data linked altered miRNA expression with exposure to toxic elements [22]; in particular it has been shown that blood leukocytes expression levels of two miRNAs involved in oxidative stress response, miR-146a and miR-222, were altered in human subjects exposed to lead and cadmium [23].

In the present study, we used a screening-based approach to investigate the association between personal levels of Ti and Zr, and miRNA expression profile in peripheral blood of 90 obese/overweight subjects. Findings suggest that obese individuals might represent a suitable population to investigate exogenous elements effects and related pathogenic mechanisms partly because of the increased particle absorption [24]. We traced Ti and Zr concentration in hair, obtaining a meaningful measure of internal dose exposure. We used a bioinformatic tool to perform an enrichment analysis to characterize the molecular pathways associated with Ti and Zr. This study have the potential to enhance the current understanding of the molecular mechanisms linked with the exposure to Ti and Zr commonly used in dental practice.

## Materials and Methods

### Study population

We recruited 90 obese/overweight subjects at the Center for Obesity and Work (IRCCS Fondazione Ca’Granda Ospedale Maggiore Policlinico) consequently from September 2010 to January 2011 as part of the SPHERE Study [25]. SPHERE is a cross-sectional study investigating the effects of particulate air pollution exposure in a population of susceptible overweight/obese subjects living in Lombardia Region. Each participant signed a written informed consent, approved by the Ethic Committee of the Fondazione Ca’Granda—Ospedale Maggiore Policlinico (approval number 1425). Each subject was asked to provide 7 ml of blood sample, for miRNA expression analysis, and a lock of hair cut next to the root in the occipital area of the head, for the elements quantification. We measured the percentage of granulocytes in each sample (Table 1).

**Table 1:** Characteristics of the study participants and exposure levels (n = 90).

Characteristics	
**Age,** *years*	51.6 ± 11.9
**Sex**	
***Male***	15 (16.7%)
***Female***	75 (83.3%)
BMI*, *Kg/m*^*2*^	32.9 ± 5.7
***Overweight (25 ≤ BMI< 30)***	34 (37.8%)
***Class I obesity (30 ≤ BMI <35)***	28 (31.1%)
***Class II obesity (35 ≤ BMI <40)***	19 (21.1%)
***Class III obesity (BMI≥ 40)***	9 (10.0%)
**Smoking habits**	
***Never smoker***	48 (53.3%)
***Ex-smoker***	26 (28.9%)
***Current smoker***	16 (17.8%)
**Metals in hair** (μg/g)	
***Titanium***	0.7 ± 0.4
***Zirconium***	0.046 ± 0.06
**Granulocyte, *%***	61.3 ± 7.6

### Hair collection, Ti and Zr quantification

Ti and Zr profile quantification was conducted by Doctor’s Data, a Clinical Laboratory Improvement Act/Amendment (CLIA)-approved laboratory, on each study participant using a standardized protocol (*Hair samples treatment protocol in* S1 File).

All hair specimens were cut from hair within 3cm from the scalp and stored at -80° in labeled Ziploc bags at room temperature. Hair samples were mailed to Doctor’s Data in the individual kits provided and treated using their laboratory analysis protocols [26, 27]. Hair samples were analyzed for Ti and Zr content using Inductively Coupled Plasma-Mass Spectrometry (ICP-MS).

### Blood collection and miRNA isolation

Peripheral blood was obtained on the day of examination by venipuncture from each participant (90 samples total). Samples were collected in PAXgene Blood RNA tubes, immediately sent to a laboratory where they were left at room temperature for 24 hours and then put at -80°C. Total RNA was extracted from whole blood with the MagMAX™-96 kit for microarrays, according to the manufacturer’s protocol (Ambion, TX), which was modified for miRNA extraction. To recover miRNAs, 1.25 volumes of isopropanol was added to the aqueous phase instead of the standard half volume. The remainder of the protocol was not altered from the original MagMAX for Microarrays protocol. The amount of RNA was quantified in an ND-1000 spectrophotometer (NanoDrop Technologies, Wilmington, DE). An Agilent 2100 BioAnalyzer (Agilent Technologies, Santa Clara, CA) was used to assess the RNA integrity from the RNA Integrity Number. The presence of low-molecular-weight RNA (5S) was also verified (data not shown).

### MicroRNA Expression Profiling

MiRNA expression was profiled with the TaqMan^®^ Low-Density Array (TLDA) (TaqMan^®^ Array Human MicroRNA A Cards Set v2.1; Life Technologies, Carlsbad, CA). Each TLDA card detects 384 features, including 377 human miRNAs, three endogenous small RNA controls (RNU6, RNU44, and RNU48, the first in quadruplicate), and a negative control (Ath-miR159a). All reactions were performed as specified in the manufacturer’s protocols. Briefly, after reverse transcription (RT) (*RNA Reverse Transcription for miRNA Expression Profiling in* S1 File), a total reaction mixture containing RT products and the TaqMan Universal PCR Master Mix (Life Technologies) was added to each line of TLDA after gentle vortexing. Each card was centrifuged and mechanically sealed with a Life Technologies sealer device. TLDAs were run in a 7900HT Fast Real-Time PCR System (Life Technologies) under the following thermal cycler conditions: 50°C for 2 minutes, 94.5°C for 10 minutes, 40 cycles of 97°C for 30 seconds, and 59.7°C for 1 minute.

Analysis of miRNA expression is detailed in supplemental material (*Analysis of miRNA expression data in* S1 file).

### Statistical analysis

Descriptive statistical data were obtained for the demographic, physical, and anthropometric variables. The mean and standard deviation (±SD) were calculated for normally distributed data. We used linear regression models to verify the association between Ti and Zr exposure and miRNA expression levels. MiRNA expression values were log_2_-transformed to achieve a normal distribution. Multivariable regression analyses adjusted for age, sex, BMI, smoking habits (current smoker, non-smoker, or ex-smoker), % of granulocytes and were applied to evaluate changes in miRNA expression in association with Ti and Zr concentrations. Due to the high number of comparisons, we applied a multiple comparison correction based on the Benjamini-Hochberg False Discovery Rate (FDR) control. A threshold of 0.10 was applied to the FDR *P* significance level to identify the set of top miRNAs. All statistical analyses were performed with SAS software, version 9.3.

## Results

### Characteristics of study participants and exposure levels

This study included 90 overweight and obese participants (16.7% men, 83.3% women), who had a mean age of 51.6 years. BMI was calculated as the weight divided by the height squared (kg/m^2^). Participants were classified by BMI as overweight (37.8%, 25 ≤ BMI< 30), class I obese (31.1%, 30 ≤BMI<35), class II obese (21.1%, 35 ≤BMI<40) and class III obese, or severe obese, (10.0%, BMI≥40). Mean BMI for 90 subjects was 32.9 ± 5.7 Kg/cm^2^ (Table 1).

Exposure mean levels and standard deviations to Ti and Zr are shown in Table 1. The participant’s mean level of Titanium in the hair was 0.7 μg/g (SD = 0.4) and the Zirconium mean level was 0.046 μg/g (SD = 0.06).

### Association of miRNA expression with hair elements levels

To determine whether there was a specific miRNA signature in association with exposure to Ti and Zr measured in the hair, we used TLDA to screen for miRNAs whose expression levels are correlated with Ti and Zr concentration. After data cleaning, we obtained 122 miRNAs (*Analysis of miRNA expression data in* S1 File). We investigated their association with Ti and Zr exposure values using multivariable regression analysis. Using an FDR linear step-up adjustment for multiple comparisons (FDR *P* < 0.1), we found 7 miRNAs (miR-99b, miR-142-5p, miR-152, miR-193a-5p, miR-323-3p, miR-335, miR-494) specifically associated with Zr levels traced in the hair (Table 2). A positive association was observed for all the miRNAs suggesting an enhancing effect of Zr on miRNA expression levels. Every increase of 1SD in Zr levels was associated with an increase in expression ranging from 35% to 58%. Even considering a broader FDR *P* threshold, we found that miRNA expression levels were still mainly associated with Zirconium levels (Table A in S1 File). Titanium levels measured in hair did not show any statistically significant association with miRNA expression levels.

**Table 2:** List of miRNAs associated with Zirconium levels.

microRNA	Exposure	%Variation	95% CI		*P*	FDR* *P*
**hsa-miR-494**	Zirconium	58.83	106.32	22.27	0.001	0.027
**hsa-miR-193a-5p**	Zirconium	35.38	63.32	12.22	0.002	0.037
**hsa-miR-142-5p**	Zirconium	40.69	74.84	13.2	0.003	0.053
**hsa-miR-335**	Zirconium	41.83	76.33	14.09	0.002	0.068
**hsa-miR-99b**	Zirconium	35.32	65.32	10.76	0.004	0.07
**hsa-miR-152**	Zirconium	43.36	82.26	12.77	0.004	0.074
**hsa-miR-323-3p**	Zirconium	50.37	95.02	15.94	0.003	0.098

*FDR: False Discovery Rate

Percentage variation express the percentage change of miRNA expression associated with an increase of 1SD in Zirconium (%Variation = ((2^β)-1)*100 and 95% CIs).

### Prediction of miRNA targets

The seven miRNAs (miR-99b, miR-142-5p, miR-152, miR-193a-5p, miR-323-3p, miR-335, miR-494) associated with Zirconium levels with FDR *P* < 0.1, were selected for downstream target prediction analysis. To explore the functional role of the investigated miRNAs and related targets, we used the Ingenuity Pathway Analysis (IPA) software (Ingenuity Systems, Redwood City, CA, USA), selecting only the miRNA-mRNA relationships that were experimentally observed and predicted with high confidence. MicroRNA Target Filter provides experimentally validated as well as predicted microRNA-mRNA interactions from TargetScan, TarBase, miRecords and Ingenuity^®^ Knowledge Base.

The analysis identified 606 mRNA that were experimentally observed or predicted with high confidence level. More specifically, we identified that miR-152, miR-494 and miR-335 had the largest number of potentially regulated mRNAs that were 254, 208 and 105 respectively. MiR-99b, miR-193a-5p, miR-322-3p and miR-142-5p respectively regulated 25, 20, 2 and 1 targets (Table B in S1 File).

### Disease and biological function analysis

The resulting target gene lists were analyzed to identify the possible diseases and biological functions significantly associated with target gene sets. In particular, we carried out the functional analysis for diseases and functions focusing the query on mechanisms involved in inflammation, skeletal and connective tissue disorders. Table 3 shows the results restricted to inflammation, skeletal and connective tissue disorders categories; for each category, we selected the top 5 diseases and functions according to the *P-value* score.

**Table 3:** Disease and biological function associated with target genes selected with IPA for 7 miRNAs.

Categories	Diseases and Functions	*P*	# Targets
**INFLAMMATION**	Inflammation of organ	5.17x10^-20^	111
	Inflammation of body region	4.25x10^-19^	97
	Inflammation of body cavity	1.06x10^-18^	79
	Connective tissue inflammation	8.41x10^-17^	88
	Chronic inflammatory disorder	8.63x10^-17^	84
**SKELETAL DISORDERS**	Congenital anomaly of musculoskeletal system development	2.21x10^-23^	69
	Cellular development, growth and proliferation of skeletal tissue	2.52x10^-22^	57
	Congenital anomaly of skeletal bone	8.34x10^-21^	48
	Craniofacial abnormality	5.34x10^-20^	44
	Development and function of skeletal system	1.49x10^-17^	45
**CONNECTIVE TISSUE DISORDERS**	Proliferation of fibroblast cell lines	7.08x10^-21^	55
	Congenital anomaly of skeletal bone	8.34x10^-21^	48
	Craniofacial abnormality	5.34x10^-20^	44
	Proliferation of fibroblasts	5.66x10^-17^	46
	Connective tissue development and function	8.41x10^-17^	88

Within the inflammation category, the most significantly involved molecules were associated with inflammation of organs, body regions and body cavities as well as connective tissue inflammation and chronic inflammatory disorders.

The enriched pathways that we identified in association with skeletal tissue disorders appeared to be largely involved in congenital anomaly of musculoskeletal system development, cellular development/growth/proliferation of skeletal tissue, congenital anomaly of skeletal bone, development and function of skeletal system and, interestingly, craniofacial abnormalities.

The enrichment analysis performed in relation to connective tissue disorders showed that target genes participates in mechanisms linked to proliferation of fibroblast cell lines, congenital anomaly of skeletal bone, craniofacial abnormality, proliferation of fibroblasts and connective tissue development.

### Pathway analysis

In the following approach, we aimed at identifying the pathways mainly regulated by the 7 miRNAs. We considered the genes that were related at least with three of the selected miRNAs (S1 Table—gene selection procedure). As expected by the large number of predicted targets, miR-152, miR-335 and miR-494 resulted to share targets such as the cytokine receptor KIT, the Rho-dependent Protein Kinase (ROCK-1), proteins belonging to the tyrosine phosphatase family (PTPN11 and PTPN14) and proteins belonging to the Rho Guanine Nucleotide Exchange Factor (ARHGEF2, ARHGEF12 and ARHGEF17).

**Fig 1:**
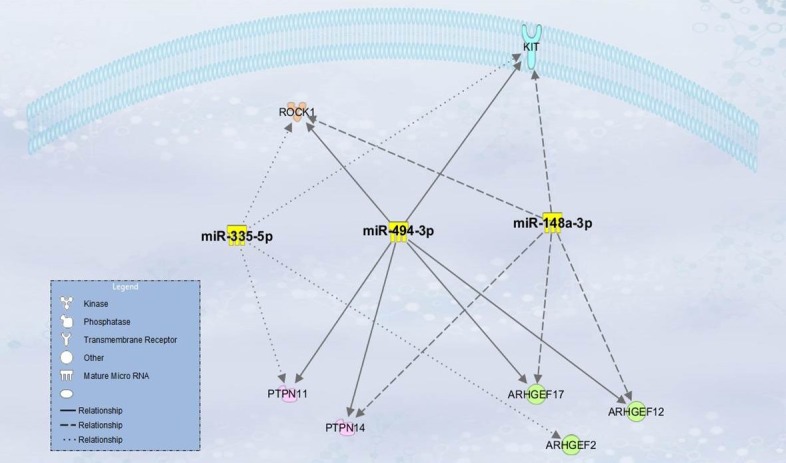
Cellular location of targets shared by miR-152, miR-335 and miR-494.

We used IPA Path Designer tool (QIAGEN) to represent the cellular location of the above mentioned targets (Fig 1) showing that their location is mainly cytoplasmic.

## Discussion

Pure Titanium, Titanium alloys and Zirconia materials are widely used in dental surgery because of their desirable mechanical properties, chemical stability, and biocompatibility [28]. In the present study on an obese population, we found that the exposure to Zr levels traced in hair is associated with a distinct signature of 7 miRNAs (miR-99b, miR-142-5p, miR-152, miR-193a-5p, miR-323-3p, miR-335, miR-494) expressed in peripheral blood. The bioinformatic analysis showed that the 7 miRNAs are largely involved in inflammation, skeletal and connective tissue disorders and target common mRNAs coding for proteins such as c-KIT, ROCK-1, PTPN and ARHGEF. We did not observe any significant association with exposure to Ti, suggesting that there might be a miRNA-specific signature in relation to Ti or Zr exposure.

We found only one in-vitro study that investigated the association between Ti and Zr exposure on miRNAs expression using a microarray approach. Palmieri et al. analyzed the expression pattern of 329 human miRNAs in human osteoblast-like cells (MG63) cultured on Grade 3 Ti and ZO_2_ ceramic disks. The study reported six up-regulated miRNAs in the cells cultivated on ZO_2_ ceramics compared to the one cultivated on Ti layer (miR-214, miR-337, miR-423, miR-339, miR-377, and miR-193b), and four down-regulated miRNAs (miR-143, miR-17-5p, miR-24, and miR-22) [29]. The same authors also looked at the specific association between Zr levels and miRNA expression data and found 18 up-regulated miRNAs (miR-337, miR-423, miR-497, miR-214, miR-377, miR-296, miR-99b, miR-193b, miR-25, miR-324, miR-518a, miR-320, miR-23b, miR-93, miR-23a, miR-422b, miR-330, miR-197) and 3 down-regulated miRNAs (miR-302c, miR-369 5p, miR-10b) [30]. Consistent with their findings, we found a positive association between Zr levels in the hair and expression of miR-99b; interestingly this miRNA was found to play a major role in regulation of cell migration after epithelial damage [31].

Among the other miRNAs expressed in association with Zr levels, we found that miR-152, miR-494 and miR-335 had hundreds of potentially regulated targets, demonstrating that they might have a relevant role in the context we are studying.

So far, few studies reported the relationship between these miRNAs and exposure to exogenous elements such as nickel sulfide [32] and lead [33]. The common targets we identified for miR-152, miR-494 and miR-335 seem to be largely involved in the regulation of inflammatory processes. C-KIT is a type III tyrosine kinase receptor with function in a diverse range of biological processes; an alteration of the role of c-kit during injury, infection and inflammation is well recognized [34]. ROCK-1 is a closely related Rho kinase that has been shown to have a specific role in the recruitment and migration of circulating inflammatory cells such as macrophages and neutrophils, both in-vitro and in-vivo [35]. PTPs proteins are critical for the regulation of fundamental cellular signaling processes; dysfunction of those PTPs brings to aberrant and uncontrolled immune responses that result in chronic inflammatory conditions. Evidence shows that expression levels of PTPN2, PTPN11, and PTPN22 are altered in actively inflamed tissues and PTPN2 seems to be critical for regulating innate and adaptive immune responses [36]. Finally the role of ARHGEF proteins have been studied in the regulation of chemotaxis of mouse macrophages, T and B lymphocytes, and bone marrow-derived mature dendritic cells [37].

Differently from the above mentioned studies, this work was conducted on an obese population samples with personal values of miRNA expression data and levels of exposure to Ti and Zr. According to the increasing need of using noninvasive markers to study the exposure to potentially toxic elements, this work focuses on the quantification of Ti and Zr in hair samples. Analysis of metals in hair has been performed for almost 150 years but only recently it has been used to identify exposures to potentially toxic elements in occupational and environmental medicine. The main advantages of this type of sampling are the simplicity of the collection and the stability during the conservation process. Moreover, differently from blood, urine or atmosphere, hair represent an internal dose of accumulation [38]. This kind of assessment provides a long term evaluation of the deposition of the element in the 3–4 months during which the hair grew.

The percent change of the 7 miRNAs we found in the present study is small, thus indicating that miRNA expression mechanism is tightly regulated. We want to address that the lack of a control group reduced our chance to observe significant changes. MiRNA changes in healthy subjects, unlike in diseased conditions, are often small and may accumulate over time, making difficult to establish the precise cause—effect relationships among exposure, miRNA alterations, and possible outcomes. Since that the obese population under this study is characterized by a wide range of BMI values, the results we obtained are not addressable to the general population. However, another strength of the present study consists on the use of real-time PCR approach which gives high precision and sensitivity in detecting the expression levels of closely related miRNAs that might differ in sequence by only one base [39]. Moreover, since the relatively small sample size might have limited our capability to detect significant effects, larger studies are needed to confirm our findings. A longitudinal study approach with multiple measures of exposures and miRNA expression at multiple times would be ideal to study miRNA dynamics in relation to Ti and Zr exposures.

## Conclusion

In conclusion, our data provide further evidence that miRNAs take part in a mechanism sensitive to Zirconium exposure. Identifying specific miRNAs will help our understanding of the biological effects of host-implant integration with the artificial devices used in dentistry and other medical therapies. The body's immediate response to a foreign object is immune-mediated reaction; there have been several attempts to embed or coat anti-inflammatory drugs and pro-regulatory molecules onto medical devices with the aim of preventing implant rejection by the host. The expression of specific miRNAs revealed several osteogenic genes as potential targets that may influence the genetic mechanisms leading to osteogenic differentiation or protect against undesired host responses. The identification of specific miRNAs will help our understanding of biological effects related to the devices used in implantology, with the ultimate aim of taking advantage of miRNAs possible therapeutic role in wound healing and host-implant integration.

## Supporting Information

S1 File (DOCX)

S1 Table (XLSX)
