# Supplementary material for: Titanium and Zirconium Levels Are Associated with Changes in MicroRNAs Expression: Results from a Human Cross-Sectional Study on Obese Population
Source: PLoS One. 2016 Sep 9;11(9):e0161916. doi: 10.1371/journal.pone.0161916 (PMC5017677; doi:10.1371/journal.pone.0161916)
Supplement: S1 File — (DOCX) [file pone.0161916.s001.docx]

**Titanium and Zirconium levels are associated with changes in microRNAs expression: results from a human cross-sectional study.**

Gianguido Cossellu, Valeria Motta, et al.

**SUPPLEMENTAL MATERIAL**

**Hair samples treatment protocol**

At Doctor’s Data, the hair specimens were further cut and washed using a modified method developed by the International Atomic Energy Agency. The hair specimens were cut into approximately 0.3 cm pieces and mixed to allow a representative sub-sampling of the hair specimen. After cutting, each sample was washed four times with a 1:200 v/v dilution of Triton X-100, then rinsed with acetone and allowed to drain. Samples were then rinsed three times with ultra-pure deionized water and two times with acetone. The dried samples were weighed prior to nitric acid/microwave digestion. After digestion, the samples were cooled and a 500 μL aliquot of an internal standard (IS) was added. Each of the resultant IS-spiked samples were diluted with 50 mL of ultrapure, deionized water.

To ensure validity, calibration verification standards, a certified hair reference control, in-house controls, spiked hair samples and other appropriate control samples were analyzed by Doctor’s Data.

**RNA reverse transcription for miRNA expression profiling**

Reverse transcription (RT) was performed using the TaqMan MicroRNA Reverse Transcription Kit and Megaplex RT Primers A following the manufacturer’s instructions (Life Technologies, CA). Briefly, 3 μL of RNAs (110 ng) from blood were added to 4.5 μL of the RT reaction mix reaction mixture including 0.8 μl Megaplex RT Primer Pools A (10×), 0.2 μl dNTPs (100 nM), 1.5 μl MultiScribe Reverse Transcriptase (50 U/μl), 0.8 μl RT Buffer (10×), 0.9 μl MgCl_2_ (25 mM), 0.1 μl RNase inhibitor (20 U/μl) and 0.2 μl nuclease-free water. After incubation on ice for 5 min reverse transcription was performed using a C1000 Thermal Cycler (Biorad, Hercules, CA). Thermal cycler conditions were as follows: 40 cycles of 16°C for 2 minutes, 42°C for 1 minute, and 50°C for 1 second, followed by 1 step of 85°C for 5 minute. The RT reaction was stored at -20°C.

**Analysis of miRNA expression data**

Real-time PCRs were used to quantify miRNA expression in terms of cycle threshold (Ct). We applied restrictive miRNA selection criteria. Values that were missing, had a Ct greater than 33, or had an ampscore less than 1 were set equal to the detection limit (35 Ct). To reduce background noise, we excluded miRNAs with at least 90% of samples not expressed (Ct = 35), producing a final dataset of 122 miRNAs. For each miRNA, the expression level was normalized to the average Ct of three endogenous controls (RNU6, RNU44, and RNU48). The fold change relative to the calibrator (pool of all 90 samples) was calculated for expression data analysis**.**

Table A: List of miRNAs in association with Zr and Ti levels.

| microRNA | Exposure | %Variation | *P* | FDR* *P* |
| --- | --- | --- | --- | --- |
| hsa-miR-494 | Zirconium | 58,83 | 0.001 | 0.027 |
| hsa-miR-193a-5p | Zirconium | 35,38 | 0.002 | 0.037 |
| hsa-miR-142-5p | Zirconium | 41,83 | 0.003 | 0.053 |
| hsa-miR-335 | Zirconium | 40,69 | 0.002 | 0.068 |
| hsa-miR-99b | Zirconium | 50,37 | 0.004 | 0.070 |
| hsa-miR-152 | Zirconium | 49,5 | 0.004 | 0.074 |
| hsa-miR-323-3p | Zirconium | 35,32 | 0.003 | 0.098 |
| hsa-miR-10a | Zirconium | 43,36 | 0.003 | 0.109 |
| hsa-miR-132 | Zirconium | 31,24 | 0.006 | 0.115 |
| hsa-miR-193a-5p | Titanium | 32,81 | 0.010 | 0.129 |
| hsa-miR-324-5p | Zirconium | 34,24 | 0.005 | 0.146 |
| hsa-miR-502-3p | Zirconium | 32,24 | 0.008 | 0.160 |
| hsa-miR-28-5p | Zirconium | 31,85 | 0.007 | 0.179 |
| hsa-miR-221 | Zirconium | 27,8 | 0.030 | 0.192 |
| hsa-miR-128 | Zirconium | 32,5 | 0.008 | 0.195 |
| hsa-miR-485-3p | Zirconium | 29,38 | 0.010 | 0.197 |
| hsa-miR-342-5p | Zirconium | 28,46 | 0.005 | 0.203 |
| hsa-miR-125a-5p | Zirconium | 40,45 | 0.012 | 0.203 |
| hsa-miR-362-3p | Zirconium | 41,41 | 0.011 | 0.205 |
| hsa-miR-148b | Zirconium | 37,18 | 0.011 | 0.211 |
| hsa-miR-495 | Zirconium | 49,99 | 0.011 | 0.215 |
| hsa-miR-130a | Zirconium | 33,13 | 0.012 | 0.234 |
| hsa-miR-27a | Zirconium | 26,41 | 0.013 | 0.250 |
| hsa-miR-340 | Zirconium | 33,56 | 0.013 | 0.252 |
| hsa-miR-181a | Titanium | 36,42 | 0.013 | 0.254 |
| hsa-miR-500 | Zirconium | 55,41 | 0.014 | 0.265 |
| hsa-miR-200c | Zirconium | 27,33 | 0.014 | 0.281 |
| hsa-miR-636 | Zirconium | 28,97 | 0.015 | 0.281 |
| hsa-miR-130b | Zirconium | 25,79 | 0.017 | 0.314 |
| hsa-miR-590-5p | Zirconium | 28,36 | 0.038 | 0.328 |
| hsa-miR-150 | Zirconium | 22,31 | 0.017 | 0.335 |
| hsa-miR-194 | Zirconium | 24,41 | 0.019 | 0.363 |
| hsa-miR-28-3p | Zirconium | 30,69 | 0.028 | 0.364 |
| has-miR-155 | Zirconium | 41,42 | 0.019 | 0.365 |
| hsa-miR-501-5p | Zirconium | 33,86 | 0.028 | 0.365 |
| hsa-miR-363 | Zirconium | 40,56 | 0.019 | 0.374 |
| hsa-miR-324-3p | Zirconium | 24,94 | 0.040 | 0.385 |
| hsa-miR-29a | Zirconium | 23,17 | 0.010 | 0.388 |
| hsa-miR-29a | Titanium | 26,85 | 0.020 | 0.388 |
| hsa-miR-125b | Zirconium | 25,22 | 0.029 | 0.395 |
| hsa-miR-29c | Zirconium | 27,17 | 0.021 | 0.404 |
| hsa-miR-744 | Zirconium | 20,44 | 0.021 | 0.417 |
| hsa-miR-18a | Zirconium | 21,45 | 0.054 | 0.419 |
| hsa-miR-491-5p | Zirconium | 28,99 | 0.022 | 0.427 |
| hsa-miR-339-3p | Zirconium | 29,59 | 0.022 | 0.431 |
| hsa-miR-101 | Zirconium | 28,73 | 0.047 | 0.455 |
| hsa-miR-133a | Zirconium | 28,39 | 0.111 | 0.459 |
| hsa-miR-139-5p | Zirconium | 20,57 | 0.036 | 0.462 |
| hsa-miR-636 | Titanium | 30,37 | 0.065 | 0.475 |
| hsa-miR-200c | Titanium | 22,16 | 0.073 | 0.478 |
| hsa-miR-574-3p | Zirconium | 25,74 | 0.075 | 0.487 |
| hsa-miR-103 | Zirconium | 27,37 | 0.026 | 0.488 |
| hsa-miR-140-5p | Zirconium | 27,5 | 0.082 | 0.494 |
| hsa-miR-197 | Zirconium | 22,3 | 0.090 | 0.504 |
| hsa-miR-328 | Zirconium | 30,75 | 0.133 | 0.530 |
| hsa-miR-423-5p | Zirconium | 21,65 | 0.097 | 0.538 |
| hsa-miR-181a | Zirconium | 17,83 | 0.041 | 0.538 |
| hsa-miR-132 | Titanium | 30,26 | 0.116 | 0.540 |
| hsa-miR-197 | Titanium | 32,38 | 0.151 | 0.540 |
| hsa-miR-148a | Zirconium | 18,83 | 0.028 | 0.550 |
| hsa-miR-422a | Zirconium | 25,84 | 0.029 | 0.556 |
| hsa-miR-103 | Titanium | 21,88 | 0.088 | 0.573 |
| hsa-miR-100 | Zirconium | 28,45 | 0.353 | 0.574 |
| hsa-miR-210 | Zirconium | 21,48 | 0.131 | 0.587 |
| hsa-miR-210 | Titanium | 23,29 | 0.133 | 0.587 |
| hsa-miR-22 | Zirconium | 28,07 | 0.045 | 0.589 |
| hsa-miR-652 | Zirconium | 33,75 | 0.065 | 0.592 |
| hsa-let-7d | Zirconium | 34,26 | 0.058 | 0.600 |
| hsa-miR-671-3p | Zirconium | 31,43 | 0.035 | 0.603 |
| hsa-let-7a | Zirconium | 24,78 | 0.060 | 0.605 |
| hsa-miR-146a | Zirconium | 24,26 | 0.125 | 0.609 |
| hsa-miR-454 | Zirconium | 29,62 | 0.145 | 0.627 |
| hsa-miR-660 | Zirconium | 22,21 | 0.063 | 0.631 |
| hsa-miR-342-5p | Titanium | 16,8 | 0.049 | 0.633 |
| hsa-miR-491-5p | Titanium | 23,1 | 0.114 | 0.633 |
| hsa-miR-598 | Zirconium | 18,97 | 0.050 | 0.645 |
| hsa-miR-99a | Zirconium | 21,58 | 0.348 | 0.645 |
| hsa-miR-886-5p | Zirconium | 21,66 | 0.050 | 0.650 |
| hsa-miR-340 | Titanium | 19,97 | 0.072 | 0.655 |
| hsa-miR-328 | Titanium | 24,06 | 0.219 | 0.656 |
| hsa-miR-629 | Zirconium | 17,57 | 0.034 | 0.657 |
| hsa-miR-195 | Zirconium | 20,28 | 0.135 | 0.658 |
| hsa-miR-486-3p | Zirconium | 17,38 | 0.182 | 0.660 |
| hsa-miR-374a | Zirconium | 15,15 | 0.068 | 0.662 |
| hsa-miR-222 | Zirconium | 16,17 | 0.160 | 0.677 |
| hsa-miR-324-5p | Titanium | 18,76 | 0.070 | 0.681 |
| hsa-miR-296-5p | Zirconium | 28,49 | 0.056 | 0.683 |
| hsa-miR-625 | Zirconium | 29,87 | 0.078 | 0.688 |
| hsa-miR-323-3p | Titanium | 18,97 | 0.089 | 0.697 |
| hsa-miR-185 | Zirconium | 17,1 | 0.179 | 0.697 |
| hsa-miR-532-3p | Zirconium | 21,77 | 0.121 | 0.702 |
| hsa-miR-148a | Titanium | 19,53 | 0.118 | 0.709 |
| hsa-miR-486-5p | Zirconium | 18,17 | 0.261 | 0.712 |
| hsa-miR-486-5p | Titanium | 14,45 | 0.270 | 0.712 |
| hsa-let-7a | Titanium | 12,92 | 0.165 | 0.716 |
| hsa-miR-422a | Titanium | 11,68 | 0.166 | 0.718 |
| hsa-miR-186 | Zirconium | 19,31 | 0.251 | 0.720 |
| hsa-miR-133a | Titanium | 21,37 | 0.494 | 0.728 |
| hsa-miR-106b | Zirconium | 18,07 | 0.156 | 0.737 |
| hsa-miR-221 | Titanium | 13,63 | 0.237 | 0.739 |
| hsa-miR-25 | Zirconium | 22,52 | 0.241 | 0.741 |
| hsa-miR-320 | Zirconium | 16,7 | 0.305 | 0.744 |
| hsa-miR-345 | Zirconium | 18,68 | 0.038 | 0.745 |
| hsa-let-7e | Zirconium | 19,38 | 0.172 | 0.746 |
| hsa-miR-590-5p | Titanium | 16,69 | 0.232 | 0.752 |
| hsa-miR-744 | Titanium | 15,26 | 0.122 | 0.753 |
| hsa-miR-339-5p | Zirconium | 17,97 | 0.271 | 0.754 |
| hsa-miR-29c | Titanium | 23,53 | 0.149 | 0.759 |
| hsa-miR-185 | Titanium | 16,12 | 0.378 | 0.762 |
| hsa-let-7d | Titanium | 22,55 | 0.158 | 0.772 |
| hsa-miR-192 | Zirconium | 18,68 | 0.088 | 0.776 |
| hsa-miR-20b | Zirconium | 14,36 | 0.100 | 0.780 |
| hsa-miR-20b | Titanium | 24,05 | 0.197 | 0.780 |
| hsa-miR-26b | Zirconium | 22,15 | 0.266 | 0.783 |
| hsa-miR-26b | Titanium | 12,09 | 0.354 | 0.783 |
| hsa-miR-99a | Titanium | 10,55 | 0.746 | 0.787 |
| hsa-miR-21 | Zirconium | 25,86 | 0.182 | 0.787 |
| hsa-miR-139-5p | Titanium | 20,3 | 0.392 | 0.789 |
| hsa-miR-671-3p | Titanium | 14,41 | 0.120 | 0.794 |
| hsa-miR-22 | Titanium | 18,12 | 0.322 | 0.797 |
| hsa-miR-140-3p | Zirconium | 14,18 | 0.252 | 0.801 |
| hsa-miR-125a-5p | Titanium | 15,82 | 0.497 | 0.802 |
| hsa-miR-125b | Titanium | 12,36 | 0.393 | 0.803 |
| hsa-miR-28-3p | Titanium | 14,83 | 0.344 | 0.805 |
| hsa-miR-10a | Titanium | 13,69 | 0.563 | 0.809 |
| hsa-miR-501-3p | Zirconium | 11,62 | 0.080 | 0.809 |
| hsa-miR-501-3p | Titanium | 21,87 | 0.083 | 0.809 |
| hsa-miR-25 | Titanium | -9,19 | 0.543 | 0.818 |
| hsa-miR-26a | Zirconium | -9,62 | 0.152 | 0.819 |
| hsa-miR-532-5p | Zirconium | 14,25 | 0.067 | 0.819 |
| hsa-miR-362-5p | Zirconium | 11,17 | 0.086 | 0.821 |
| hsa-miR-324-3p | Titanium | 16,78 | 0.386 | 0.823 |
| hsa-miR-130b | Titanium | 27,75 | 0.172 | 0.828 |
| hsa-miR-100 | Titanium | 19,17 | 0.765 | 0.829 |
| hsa-miR-574-3p | Titanium | 14,96 | 0.380 | 0.830 |
| hsa-miR-128 | Titanium | 11,85 | 0.085 | 0.833 |
| hsa-miR-423-5p | Titanium | 15,97 | 0.257 | 0.834 |
| hsa-miR-486-3p | Titanium | 14,33 | 0.420 | 0.836 |
| hsa-miR-24 | Zirconium | 16,39 | 0.118 | 0.840 |
| hsa-miR-93 | Zirconium | 16,25 | 0.199 | 0.844 |
| hsa-let-7e | Titanium | 10,95 | 0.323 | 0.845 |
| hsa-miR-183 | Zirconium | 15,62 | 0.142 | 0.849 |
| hsa-miR-374b | Zirconium | 11,93 | 0.192 | 0.850 |
| hsa-miR-532-3p | Titanium | 15,4 | 0.393 | 0.850 |
| hsa-miR-495 | Titanium | 10,42 | 0.214 | 0.857 |
| hsa-let-7g | Zirconium | 16,15 | 0.233 | 0.867 |
| hsa-let-7g | Titanium | 14,08 | 0.327 | 0.867 |
| hsa-miR-320 | Titanium | 10,43 | 0.774 | 0.867 |
| hsa-miR-222 | Titanium | 9,07 | 0.293 | 0.871 |
| hsa-miR-501-5p | Titanium | 12,59 | 0.422 | 0.875 |
| hsa-miR-500 | Titanium | 12,33 | 0.207 | 0.876 |
| hsa-miR-150 | Titanium | 15,3 | 0.163 | 0.877 |
| hsa-miR-146a | Titanium | 13,56 | 0.274 | 0.877 |
| hsa-miR-192 | Titanium | 12,42 | 0.699 | 0.881 |
| hsa-miR-301a | Zirconium | 15,51 | 0.226 | 0.881 |
| hsa-miR-146b-5p | Zirconium | 17,27 | 0.052 | 0.882 |
| hsa-miR-146b-5p | Titanium | 11,03 | 0.270 | 0.882 |
| hsa-miR-335 | Titanium | 8,37 | 0.091 | 0.883 |
| hsa-miR-142-3p | Zirconium | 8,59 | 0.175 | 0.885 |
| hsa-miR-502-3p | Titanium | 7,44 | 0.149 | 0.886 |
| hsa-miR-342-3p | Zirconium | 17,38 | 0.584 | 0.887 |
| hsa-miR-342-3p | Titanium | 11,88 | 0.775 | 0.887 |
| has-miR-155 | Titanium | 7,77 | 0.152 | 0.888 |
| hsa-miR-362-3p | Titanium | 10,81 | 0.092 | 0.892 |
| hsa-miR-598 | Titanium | 11,25 | 0.216 | 0.895 |
| hsa-miR-182 | Zirconium | 7,16 | 0.310 | 0.895 |
| hsa-miR-182 | Titanium | 11,28 | 0.784 | 0.895 |
| hsa-miR-30b | Zirconium | 9,12 | 0.686 | 0.896 |
| hsa-miR-362-5p | Titanium | 15,96 | 0.146 | 0.897 |
| hsa-miR-485-3p | Titanium | 14,74 | 0.281 | 0.899 |
| hsa-miR-191 | Titanium | 10,82 | 0.548 | 0.903 |
| hsa-miR-191 | Zirconium | 13,27 | 0.671 | 0.903 |
| hsa-miR-363 | Titanium | 7,02 | 0.187 | 0.903 |
| hsa-miR-19a | Zirconium | 11,53 | 0.283 | 0.904 |
| hsa-miR-99b | Titanium | 8,02 | 0.585 | 0.904 |
| hsa-miR-451 | Titanium | 13,12 | 0.407 | 0.906 |
| hsa-miR-451 | Zirconium | -8,48 | 0.680 | 0.906 |
| hsa-miR-20a | Zirconium | -5,8 | 0.232 | 0.906 |
| hsa-miR-16 | Titanium | 9,17 | 0.690 | 0.907 |
| hsa-miR-17 | Zirconium | 9,12 | 0.454 | 0.907 |
| hsa-miR-17 | Titanium | 9,94 | 0.509 | 0.907 |
| hsa-miR-28-5p | Titanium | 8,87 | 0.164 | 0.909 |
| hsa-miR-625 | Titanium | 20,84 | 0.386 | 0.911 |
| hsa-miR-106b | Titanium | 9,89 | 0.442 | 0.914 |
| hsa-miR-142-3p | Titanium | 12,43 | 0.738 | 0.921 |
| hsa-miR-130a | Titanium | 11,2 | 0.181 | 0.921 |
| hsa-miR-339-5p | Titanium | 7,18 | 0.536 | 0.922 |
| hsa-miR-425 | Titanium | 8,71 | 0.639 | 0.923 |
| hsa-miR-331-3p | Zirconium | 10,03 | 0.735 | 0.925 |
| hsa-miR-194 | Titanium | 9,11 | 0.325 | 0.928 |
| hsa-miR-15b | Zirconium | 8,58 | 0.377 | 0.928 |
| hsa-miR-15b | Titanium | 7,22 | 0.561 | 0.928 |
| hsa-miR-301a | Titanium | 6,05 | 0.485 | 0.928 |
| hsa-miR-106a | Zirconium | 8,51 | 0.451 | 0.929 |
| hsa-miR-106a | Titanium | 10,39 | 0.579 | 0.929 |
| hsa-miR-195 | Titanium | -5,5 | 0.297 | 0.938 |
| hsa-miR-425 | Zirconium | 6,14 | 0.746 | 0.939 |
| hsa-miR-223 | Titanium | -4,78 | 0.283 | 0.940 |
| hsa-miR-223 | Zirconium | 5,39 | 0.440 | 0.940 |
| hsa-miR-345 | Titanium | 8,2 | 0.394 | 0.940 |
| hsa-miR-886-5p | Titanium | 11,21 | 0.408 | 0.942 |
| hsa-miR-24 | Titanium | 11,49 | 0.378 | 0.942 |
| hsa-miR-183 | Titanium | 7,96 | 0.554 | 0.943 |
| hsa-miR-454 | Titanium | 8,94 | 0.385 | 0.950 |
| hsa-miR-101 | Titanium | 10,5 | 0.361 | 0.950 |
| hsa-miR-339-3p | Titanium | 9,25 | 0.451 | 0.950 |
| hsa-miR-19b | Titanium | 7,22 | 0.518 | 0.951 |
| hsa-miR-19b | Zirconium | 6,42 | 0.889 | 0.951 |
| hsa-miR-652 | Titanium | 7,88 | 0.745 | 0.951 |
| hsa-miR-532-5p | Titanium | 20,54 | 0.327 | 0.954 |
| hsa-miR-374b | Titanium | -4,71 | 0.550 | 0.954 |
| hsa-miR-30c | Titanium | 3,24 | 0.938 | 0.955 |
| hsa-miR-30c | Zirconium | -7,51 | 0.955 | 0.955 |
| hsa-miR-193b | Titanium | 3,7 | 0.067 | 0.956 |
| hsa-miR-193b | Zirconium | 16,84 | 0.299 | 0.956 |
| hsa-miR-20a | Titanium | 3,53 | 0.405 | 0.958 |
| hsa-miR-660 | Titanium | 18,54 | 0.516 | 0.958 |
| hsa-miR-296-5p | Titanium | 2,77 | 0.256 | 0.960 |
| hsa-miR-21 | Titanium | 3,13 | 0.524 | 0.960 |
| hsa-miR-484 | Zirconium | 3,09 | 0.443 | 0.961 |
| hsa-miR-93 | Titanium | -3,53 | 0.444 | 0.962 |
| hsa-miR-484 | Titanium | 2,35 | 0.536 | 0.963 |
| hsa-miR-374a | Titanium | 5,92 | 0.425 | 0.964 |
| hsa-miR-186 | Titanium | 12,44 | 0.727 | 0.968 |
| hsa-miR-26a | Titanium | 5,38 | 0.443 | 0.968 |
| hsa-miR-331-3p | Titanium | -5,7 | 0.965 | 0.969 |
| hsa-miR-140-5p | Titanium | 2,31 | 0.345 | 0.970 |
| hsa-miR-92a | Titanium | 4,05 | 0.896 | 0.971 |
| hsa-miR-494 | Titanium | 2,25 | 0.222 | 0.971 |
| hsa-let-7b | Zirconium | 2,8 | 0.620 | 0.972 |
| hsa-let-7b | Titanium | -5,29 | 0.954 | 0.972 |
| hsa-miR-142-5p | Titanium | -2,06 | 0.149 | 0.974 |
| hsa-miR-19a | Titanium | -5,61 | 0.783 | 0.975 |
| hsa-miR-152 | Titanium | 1,18 | 0.102 | 0.977 |
| hsa-miR-27a | Titanium | 4,61 | 0.119 | 0.977 |
| hsa-miR-140-3p | Titanium | -0,56 | 0.816 | 0.979 |
| hsa-miR-30b | Titanium | 0,59 | 0.968 | 0.981 |
| hsa-miR-16 | Zirconium | 0,41 | 0.981 | 0.981 |
| hsa-miR-145 | Zirconium | 2,53 | 0.115 | 0.986 |
| hsa-miR-145 | Titanium | -0,35 | 0.524 | 0.986 |
| hsa-miR-92a | Zirconium | 2,83 | 0.991 | 0.991 |
| hsa-miR-18a | Titanium | -0,3 | 0.324 | 0.993 |
| hsa-miR-148b | Titanium | 5,23 | 0.087 | 0.996 |
| hsa-miR-126 | Zirconium | -0,29 | 0.473 | 0.997 |
| hsa-miR-126 | Titanium | -0,99 | 0.764 | 0.997 |
| hsa-miR-629 | Titanium | 0,08 | 0.420 | 0.997 |

Table B: number of targets for each selected miRNA.

| miRNA | # of targets |
| --- | --- |
| hsa-miR-142-5p | 1 |
| hsa-miR-152 | 254 |
| hsa-miR-193a-5p | 20 |
| hsa-miR-323-3p | 2 |
| hsa-miR-335 | 105 |
| hsa-miR-494 | 208 |
| hsa-miR-99b | 25 |
|  | 615 |
